# Deciphering molecular properties of hypermutated gastrointestinal cancer

**DOI:** 10.1111/jcmm.13941

**Published:** 2018-10-31

**Authors:** Wangxiong Hu, Yanmei Yang, Weiting Ge, Shu Zheng

**Affiliations:** ^1^ Cancer Institute (Key Laboratory of Cancer Prevention and Intervention, China National Ministry of Education) The Second Affiliated Hospital Zhejiang University School of Medicine Zhejiang China; ^2^ Key Laboratory of Reproductive and Genetics Ministry of Education Women's Hospital Zhejiang University School of Medicine Zhejiang China

**Keywords:** hypermethylation, hypermutation, mismatch repair system

## Abstract

Great mutational heterogeneity is observed both across cancer types (>1000‐fold) and within a given cancer type, with a fraction harboring >10 mutations per million bases, thus termed hypermutation. We determined the genome‐wide effects of high mutation load on the transcriptome and methylome of two cancer types; namely, colorectal cancer (CRC) and stomach adenocarcinoma (STAD). Briefly, hierarchical clustering of the expression and methylation profiles showed that the majority of CRC and STAD hypermutated samples were mixed and separated from their respective non‐hypermutated samples, exceeding the boundary of tissue specificity. Further in‐detailed exploration uncovered that the underlying molecular mechanism may be related to the perturbation of chromatin remodeling genes.

## 1 INTRODUCTION

A tumor is largely caused by somatic mutations.^1^, ^2^ In general, mutation of a few genes is sufficient to initiate tumorigenesis.^1^ However, great mutational heterogeneity is observed both across cancer types (>1000‐fold) and within a given cancer type.^3^ The wide range of mutation frequency within a given cancer type (eg, in melanoma and lung cancer, the frequency ranged between 0.1‐100/million bases [Mb]) has prompted researchers to classify cancers into hypermutated and non‐hypermutated forms.^4^, ^5^

Commonly, a sample is defined as hypermutated if its mutation rate is >10 mutations per Mb.^4^, ^6^ Hypermutation can be caused by in vitro (environmental mutagen exposure such as UV light and tobacco smoke) and in vivo factors (dysregulation of apolipoprotein B mRNA editing enzyme, catalytic polypeptide‐like (APOBEC) family members and defects in DNA polymerase ε (*POLE*), Polδ1 (*POLQ*1), or the DNA mismatch repair system (MMR)).^4^, ^7^, ^8^ Tumors with a large number of somatic mutations may be susceptible to immune checkpoint blockade (eg, PD‐1).^9^, ^10^, ^11^ Thus, microsatellite instability‐high (MSI‐H) status (commonly linked to high mutation burden) may be associated with a better prognosis.^12^, ^13^ However, the transcriptome and epigenetic profiles, such as the DNA methylome, in hypermutated samples are poorly understood. Here, we focused on two cancer types (CRC and STAD) with a relatively high proportion of intrinsic hypermutation.

## 2 MATERIALS AND METHODS

### 2.1 Hypermutation definition

All cancer somatic mutation data and clinical information were downloaded from The Cancer Genome Atlas (TCGA) data portal (11/03/2015). Silent mutations and RNA mutations were discarded from further analyses. The hypermutated criterion was defined, which was similar to the work by Campbell et al.^4^ Briefly, the breakpoint at which a significant change in the slope occurred was determined to be the hypermutation threshold. Thus, the samples with over 10 mutations per Mb were defined as hypermutated.

### 2.2 Gene expression data processing and normalization

All level 3 tumor mRNA expression data sets (RNASeqV2) were obtained from the TCGA (October 2015). Known batch effects were corrected using the *ComBat* function in the Bioconductor *sva* package.^14^ Analysis of differentially expressed mRNA between hypermutated and non‐hypermutated was performed using the *DEGSeq* package for R/Bioconductor.^15^ Genes with expression levels <1 (RNA‐Seq by Expectation Maximization (RSEM)‐normalized counts) in more than 50% of samples were removed. Significant differentially expressed mRNAs were selected according to a false discovery rate (FDR) adjusted *P* value <0.05 and fold change >2 conditions.

### 2.3 HM450k data retrieval and process

CRC and STAD level three DNA methylation data (HumanMethylation450) were downloaded from the TCGA data portal (11/13/2016). The methylation level of each probe was measured with a beta value ranging from 0 to 1; this is calculated as the ratio of the methylated signal to the sum of the methylated and unmethylated signals. Probes with an “NA” value in more than 10% of the CRC or STAD samples were discarded. Next, the *limma* Bioconductor package was used to identify differentially methylated sites (DMSs) in the remaining probes.^16^ Significant DMSs were selected according to the FDR adjusted *P* value <1E‐20 condition. All heatmaps were generated using the *pheatmap* package in R (64‐bit, version 3.0.2).

### 2.4 Survival analysis

Genes that correlated with patient survival time in the multivariate Cox regression analysis were determined using the least absolute shrinkage and selection operator (LASSO) method. The best *λ* was determined by 10‐fold cross‐validation using the *glmnet* package built‐in function *cv.glmnet*.^17^ For each group, we divided the patients into high‐ and low‐risk groups by calculating the prognostic index (PI) as follows:

$${\text{PI}}_{k}=\sum_{g=1}^{n}\beta_{g}m_{\mathrm{gk}}$$

where n is the number of survival correlated genes, *β*_*g*_ is the regression coefficient of the Cox proportional hazard model for gene *g*, and *m*_*gk*_ is the expression level of gene *g* in patient *k*. Patients were then divided into high‐ and low‐risk groups based on the median PI. The survival difference between the two groups (good‐ and poor‐prognosis) was tested by the Kaplan‐Meier method and analyzed with the log‐rank test with functions *survfit* and *survdiff* in the *survival* package for R.^18^ A *P* value <0.05 was considered significant.

### 2.5 Hub gene definition

Co‐expression network construction was performed as described in our previous work.^19^ Hub genes were those with an extremely high level of connectivity in a given network. Connectivity reflects how frequently a node interacts with other nodes and the sum of the weights across all edges of a node. Because some modules (also known as networks) were rather large, we restricted the number of genes in the output of the module. Here, the top 50 genes with the highest connectivity in each network that were reasonable to display were defined as hub genes as previously described.^20^

### 2.6 Data and code availability

Codes used in this study are available at https://github.com/huwangxiong/Hypermutated_gastrointestinal_cancer (Digital Object Identifier: https://doi.org/10.5281/zenodo.1406067). All other data are available from the authors upon request.

## 3 RESULTS

### 3.1 Characteristics of hypermutated samples in CRC and STAD

Consistent with the observation by Campbell et al^4^, here, patients with over 10 mutations per Mb were defined as hypermutated (Figure S1A and B). This outcome established 61 (16%) and 83 (24%) of the samples as hypermutated in CRC and STAD, respectively. Of the 61 hypermutated samples in CRC, no difference was observed in age and gender in comparison with the 322 non‐hypermutated samples. However, 77% of the hypermutated samples originated from patients who were diagnosed with stage I/II, while an even distribution was observed in the non‐hypermutated group (Table 1). Notably, all hypermutated CRC samples were classified as MSI‐H using the criteria of Hause et al^21^, and 80% were located in the right side of the colon. In STAD, 60% of the hypermutated cases were median‐aged (60‐75), and no significant difference was observed in gender when compared with the non‐hypermutated samples. Notably, no stage prevalence was observed in the STAD hypermutated samples, which was in contrast with the CRC samples. However, similar to the findings in CRC, 84% of the hypermutated cases were driven by MSI‐H with microsatellite status annotation (Table 1).

**Table 1:** Clinical characteristics of hypermutated and non‐hypermutated in colorectal cancer (CRC) and stomach adenocarcinoma (STAD)

Variable	CRC			STAD		
	Hyper	Non‐hyper	*P*	Hyper	Non‐hyper	*P*
	n = (61)	n = (322)		n = (72)	n = (268)	
Age						
<60	19	94	0.1343	11	96	0.00379
60‐75	22	153		44	129	
>75	21	75		16	41	
Unknown	0	0		1	2	
Sex						
Male	38	181	0.4597	37	180	0.01954
Female	23	141		35	88	
Stage						
I	10	51	<0.001	15	34	0.2178
II	37	103		23	85	
III	11	108		23	116	
IV	2	44		7	25	
Unknown	1	16		4	8	
MMR						
MSI‐H	61	0	<0.001	31	0	<0.001
MSS or MSI‐L	0	322		6	157	
Unknown	0	0		36	111	
Location						
Left	11	205	<0.001			
Right	45	105				
Unknown	5	12				

In the hypermutated tumors, *ARID1A*,* RNF43*,* LRP1B*,* FAT1˜4*,* MLL1˜4* and *MACF1* were frequent targets of mutation in both CRC and STAD. We found that 630 and 582 genes were mutated (>20% hypermutated patients) in CRC and STAD, respectively, with 351 mutated genes shared by them.

### 3.2 Mutation‐driven expression patterns are consistent across organs

To decipher the mutation‐driven expression profiles across organs, we first compared the transcriptomes between the hypermutated and non‐hypermutated samples in CRC and STAD. In total, 935 and 1047 differentially expressed genes (DEGs) were identified in CRC (hypermutated vs. non‐hypermutated) and STAD (hypermutated vs. non‐hypermutated), respectively, with 185 DEGs common to both comparisons. Intriguingly, only 56 genes (5%) were upregulated in the hypermutated subgroup in STAD. Notably, the upregulated genes such as *AIM2* and *TNFRSF9* in the hypermutated samples were closely associated with immune response. AIM2 suppresses tumor growth via inhibiting AKT, which is a promising avenue for therapy or prevention.^22^, ^23^ TNFRSF9 is a critical mediator of sterile inflammation and is also a therapeutic target for cancer.^24^

**Figure 1:**
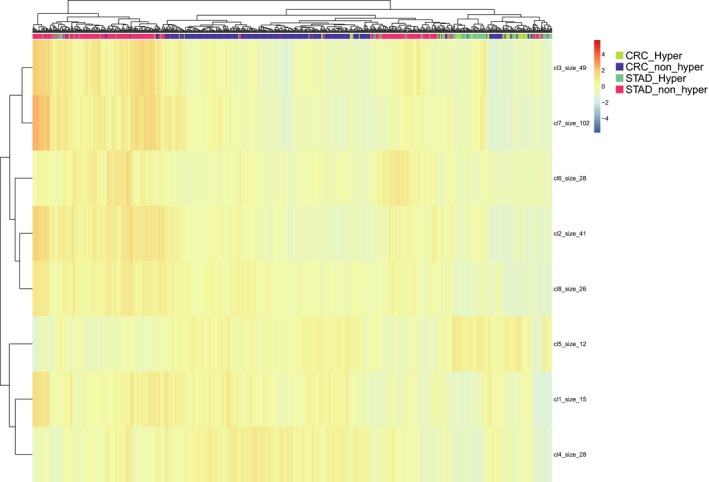
Global molecular pattern defined by hypermutation is consistent across colorectal cancer (CRC) and stomach adenocarcinoma (STAD). Heatmap depicting mRNA expression of DEGs between hypermutated and non‐hypermutated CRC. Hierarchical clustering reveals that majority of CRC and STAD hypermutated samples were mixed and separated from their respective non‐hypermutated samples

Next, we evaluated if the mutation‐driven gene expression pattern was similar across organs with DEGs identified in CRC and STAD. The results showed that most of the CRC and STAD hypermutated samples were mixed and separated from their respective non‐hypermutated counterparts, exceeding the boundary of tissue‐specificity (Figure 1).

### 3.3 Mutation‐driven methylation patterns are similar across organs

**Figure 2:**
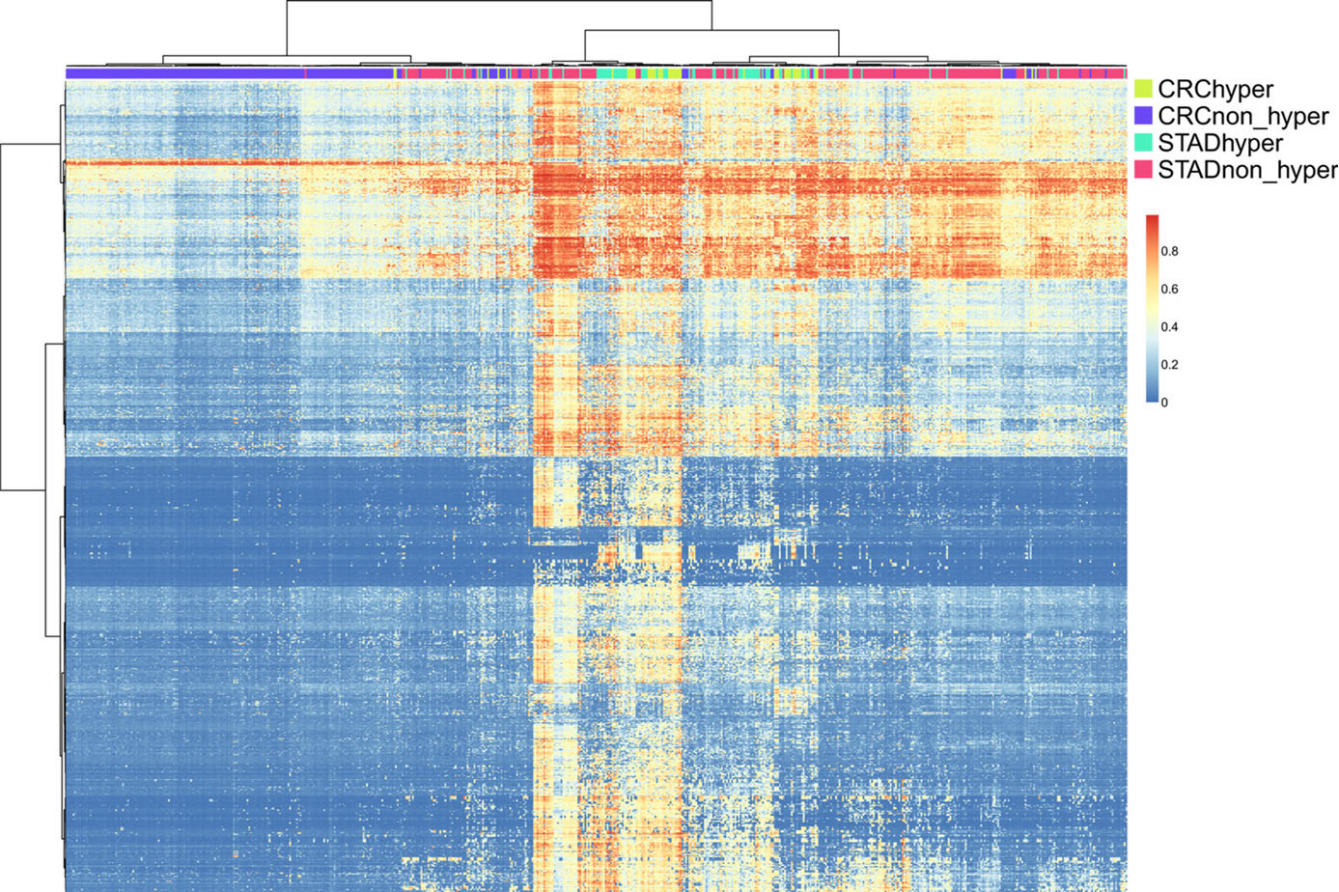
Overall DNA methylation pattern defined by hypermutation is consistent across colorectal cancer (CRC) and stomach adenocarcinoma (STAD). Heatmap depicting DNA methylation of differentially methylated sites between hypermutated and non‐hypermutated CRC. Hierarchical clustering reveals that majority of CRC and STAD hypermutated samples were mixed and separated from their respective non‐hypermutated samples

Gene expression lies in the downstream cascade of gene mutation. To further consolidate the molecular similarities in the hypermutated samples with high mutational burden across cancer types, we explored the epigenetic patterns in the hypermutated and non‐hypermutated samples between CRC and STAD. Notably, CRC was more sensitive to DNA methylation variation compared to STAD. In total, 1604 and 53 differentially methylated sites (DMSs, *P* < 1E‐20) were identified in CRC (hypermutated vs. non‐hypermutated) and STAD samples (hypermutated vs. non‐hypermutated), respectively, with 21 CpG sites common to both comparisons. Notably, 19 of the 21 common CpG sites were within *MLH1*, a gene closely associated with MSI‐H if it is inactivated. Hierarchical clustering of DNA methylation profiles showed that most CRC and STAD hypermutated samples were mixed together and separated from their respective non‐hypermutated samples, which is consistent with the expression pattern observations (Figure 2).

### 3.4 Mutation‐driven consistent methylation pattern is independent of *MLH1* hypermethylation

Inactivation of the MMR pathway is often caused by hypermethylation of the *MLH1* gene promoter.^25^ Thus, the consistent methylation pattern observed in the hypermutated groups may largely be a consequence of *MLH1* hypermethylation. Indeed, 57% (27 samples) CRC and 58% (45 samples) STAD hypermutated cases were *MLH1* hypermethylated. After excluding all *MLH1* CG probes and reclustering the methylation profiles, we found that the consistent mutation‐driven methylation patterns held (Figure S2). This result strongly suggested that consistent mutation‐driven methylation patterns in CRC and STAD were based on an unknown molecular mechanism, irrespective of *MLH1* methylation status.

### 3.5 Identifying genes and methylation probes associated with survival in STAD hypermutated samples

We next sought to find genes and CG probes that may serve as prognostic markers in hypermutated and non‐hypermutated samples. Since the power of detecting prognostic genes mainly depends on the sample size and the number of survival events (that is, death), here, we focused on STAD because the CRC hypermutated samples only contained 11 survival events. Cox proportional hazard survival analysis showed that 1851 and 1543 genes correlated with survival outcomes in STAD hypermutated and non‐hypermutated samples, respectively, with only 135 genes in common between them. This result suggested that most of the survival factors were hypermutated‐driven, not shared by non‐hypermutated cases. Genes whose increased expression was correlated with worsened survival outcomes in hypermutated samples included *EPHA5* (Ephrin Type‐A Receptor 5) (hazard ratio (HR) = 1.33, 95% confidence interval (CI) 1.178‐1.501, *P* = 4.152E‐06) and *TAGLN3* (Transgelin 3) (HR = 1.546, 95% CI 1.24‐1.929, *P* = 0.0001094). In contrast, genes whose expression was correlated with a better prognosis included *GAS2L1* (GAS2‐like protein 1) (HR = 0.9983, 95% CI 0.9974‐0.9993, *P* = 0.0004428), *PRICKLE3* (Prickle Planar Cell Polarity Protein 3) (HR = 0.9922, 95% CI 0.988‐0.9964, *P* = 0.0002996) and *RNH1* (ribonuclease/angiogenin inhibitor 1) (HR = 0.9986, 95% CI 0.998‐0.9993, *P* = 0.0001094). Additionally, we sought to determine an expression signature that could separate the hypermutated patients into two groups with either a high or low prognostic index (PI, see methods for more detail) since canonical survival‐related factors such as TNM staging and MMR status were invalid in this context. Notably, STAD hypermutated patients could be separated into two groups with high or low PI based on 1‐8 survival‐related genes (Table 2). Particularly for *RNH1* and *TAGLN3*, the discrimination power (C‐index value) reached >0.7 with only a single variable (Figure S3, Table 2). A well‐conceived five‐gene signature (*DNAI2*,* EPHA5*,* GAS2L1*,* RNH1*,* TAGLN3*) was identified that could predict prognosis of hypermutated STAD patients with high performance (C‐index: 0.84), but the robustness should be validated in another independent dataset in the future. These survival‐related genes can be prognostic signatures used in hypermutated STADs, but they warrant further validation to test whether they are broadly applicable.

**Table 2:** Expression signature that could distinguish hypermutated STAD patients into two groups with high and low prognostic index

Model	Gene number	C‐index	logrank	coxph_Wald test
DNAI2‐EPHA5‐GAS2L1‐HCG4P6‐PDE5A‐PI4KAP2‐RNH1‐TAGLN3	8	0.846702	4.15E‐05	2.99E‐09
DNAI2‐EPHA5‐GAS2L1‐RNH1‐TAGLN3	5	0.8404635	4.15E‐05	1.49E‐08
EPHA5‐GAS2L1‐RNH1‐TAGLN3	4	0.8163993	4.15E‐05	1.04E‐07
EPHA5‐GAS2L1‐RNH1	3	0.8074866	9.14E‐05	7.40E‐07
GAS2L1‐RNH1	2	0.7816399	5.81E‐06	1.55E‐05
RNH1	1	0.7593583	1.08E‐04	6.99E‐05
TAGLN3	1	0.7027629	6.50E‐03	1.09E‐04

Meanwhile, 185 and 1616 CG probes correlated with survival outcomes were identified in hypermutated and non‐hypermutated samples, respectively, with only 8 probes (cg04431629‐*DMRTA2*, cg05542757‐*FREM2*, cg10439246, cg12630461‐*RCCD1*, cg12837869, cg14696334‐*RCCD1*, cg17219660‐*GPR37L1* and cg18347921‐*NCRNA00171*) in common between them. Unlike the expression variables, we could not determine a threshold that could separate hypermutated patients into two groups with either a high or low PI. Some explanations may account for this. First, the hypermutated sample size is too small to pick a suitable model. Second, as a binary marker (methylated or hypomethylated), the methylation level cannot sensitively reflect the clinical outcome.

### 3.6 Identification of candidate therapeutic target genes in STAD hypermutated samples

**Figure 3:**
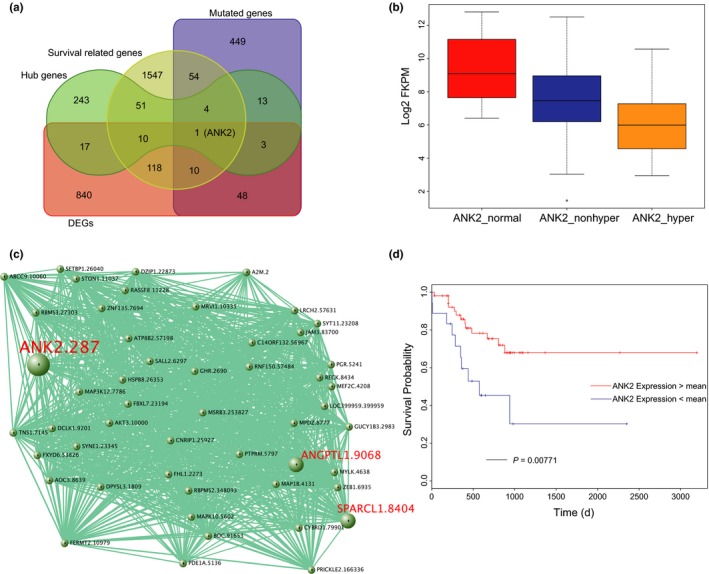
ANK2 is a candidate therapeutic target gene in stomach adenocarcinoma (STAD) hypermutated samples. A, Venn diagram of mutated, differentially expressed, survival related and act as the hub node in a regulatory network identified ANK2 as a potential therapeutic target in the treatment of hypermutated STAD patients. B, *ANK2* was highly depressed in hypermutated samples compared with non‐hypermutated and adjacent normal tissue (*P* < 0.05, Mann‐Whitney test). C, WGCNA exploration uncovered *ANK2* as a hub gene associated with some validated tumor suppressors. D, Higher *ANK2* expression level is correlated with a significantly better clinical outcome (*P* = 0.00771, logrank test)

A mutated gene that is differentially expressed, survival related and acts as a hub node in a regulatory network may serve as a potential therapeutic target in the treatment of hypermutated patients. Notably, we found only one gene, *ANK2* (Ankyrin‐2) that may serve as a potential therapeutic target in the treatment of hypermutated STADs (Figure 3A). *ANK2* was highly repressed in hypermutated samples compared with non‐hypermutated and adjacent normal tissue (*P* < 0.05, Mann‐Whitney test, Figure 3B). Co‐expression network analysis revealed that *ANK2* was a hub gene associated with some validated tumor suppressors (Figure 3C). For example, ANGPTL1 and SPARCL1, crosstalking with ANK2, have been shown to attenuate colorectal cancer metastasis in our previous work.^26^, ^27^ Hypermutated patients with higher *ANK2* expression levels had a significantly better clinical outcome (*P* = 0.00771, log‐rank test, Figure 3D). Currently, there are no ANK2‐targeting molecules available in the clinic or even pre‐clinical research. But we hope we and someone else will verify this hypothesis using in vivo activation of endogenous target genes through trans‐epigenetic remodeling in future work.^28^ The overall 5‐year survival rates were 68% (95% CI 54% to 85%) compared to 30% (95% CI 12% to 80%) based on the mean expression cutoff in hypermutated STADs.

### 3.7 Chromatin remodeling genes may be responsible for the consistent expression and methylation pattern in hypermutated samples

**Figure 4:**
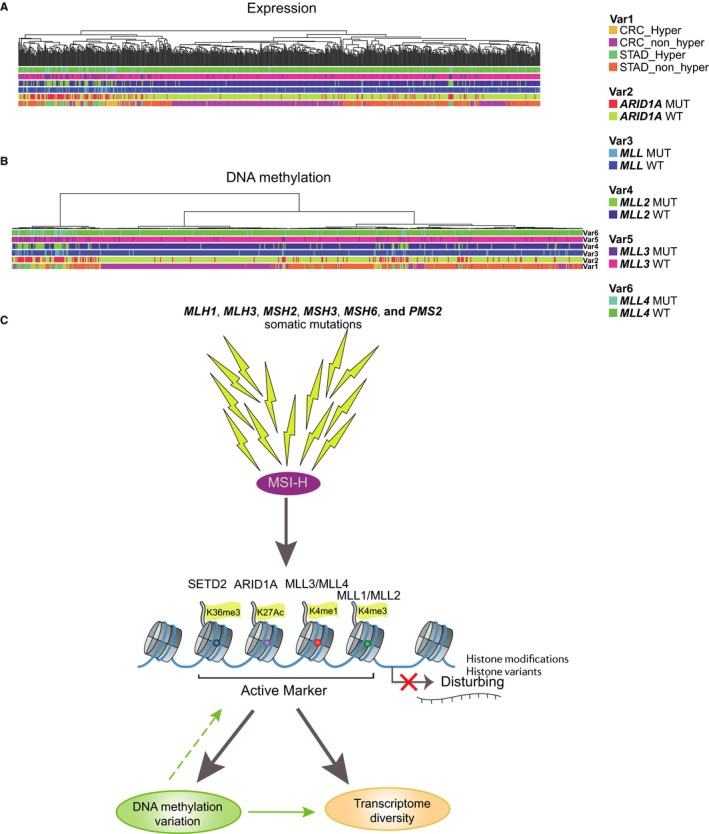
Association of chromatin remodelling genes and consistent expression and methylation pattern in hypermutated samples. A, Hierarchical clustering of DEGs with the annotation of chromatin remodeling gene (*ARID1A*,*MLL1‐4*) status reveals that chromatin remodeling genes are responsible for the consistent expression pattern in hypermutated samples. B, Hierarchical clustering of DMSs with the annotation of chromatin remodeling gene (*ARID1A*,*MLL1‐4*) status reveals that chromatin remodeling genes are responsible for the consistent methylation pattern in hypermutated samples. C, Schematic diagram of the putative molecular mechanism underlying the consistent expression and methylation pattern

The intrinsic molecular similarity shown in high mutational load samples with different tissues of origin prompted us to seek potential drivers. Correlation analysis of mutational profiles, transcriptomes and methylomes showed that inactivation of chromatin remodeling genes (*ARID1A* and *MLL1˜4*) may account for the consistent expression (Figure 4A) and methylation (Figure 4B) patterns in hypermutated samples. *ARID1A* fits the hypothesis well; it is mutated in 49% of CRC and 76% of STAD hypermutated samples but rarely exists in non‐hypermutated cases. This result was shown for *MLL2* as well. We thus concluded that an MSI‐H→chromatin remodeling genes (*ARID1A* and *MLL1˜4*) inactivation→DNA methylation/expression variation axis (Figure 4C) shared in CRC and STAD hypermutated samples may be associated with the consistent expression and methylation patterns identified in this study.

## 4 DISCUSSION

The consistent expression and DNA methylation patterns in hypermutated samples uncover a somatic driver foregoing tissue specificity. In CRC and STAD, hypermutation is largely contributed by MSI‐H. It is well‐known that MSI‐H is caused by inactivation (mutation or hypermethylation) of *MLH1*,* MLH3*,* MSH2*,* MSH3*,* MSH6* and *PMS2*.^6^ In addition, the frequent mutation of chromatin remodeling genes (*ARID1A*,* CHD6*,* CREBBP*,* SETD1A*,* NCOR1* and *MLL1˜4*) indicated that they were also putative cellular drivers in the hypermutated subgroup. To test this possibility, we examined six STAD hypermutated samples that were microsatellite stable (MSS) to exclude MSI confounding. The result showed that five of these samples were probably driven by *MLL* or *MLL3*, which was consistent with the results of previous studies.^29^, ^30^ The consistent expression and methylation patterns observed in this study raised the possibility that the DEGs were a result of the somatic mutations. To test this theory, we explored the correlation of gene mutations and DEGs in the hypermutated subgroups. In total, only 30 and 62 DEGs were associated with mutations in CRC and STAD, respectively. Rare DEGs associated with mutations revealed another molecular mechanism responsible for the expression variation in the hypermutated samples. Recently, Mathur et al^31^, found that ARID1A loss impairs enhancer‐mediated gene regulation through altering H3K27ac levels at enhancers, which led to the dysregulation of more than 1000 genes (>2‐fold). Additionally, depleting mouse ESCs of H3K4me3 writers (ie, MLL1 and MLL2) can regulate distinct subsets of genes and/or are involved in biological functions.^32^ H3K4me1 is mainly deposited by protein complexes containing MLL3 and MLL4. H3K4me3 and H3K4me1 are active markers, and their depletion may explain the downregulation of most DEGs in STAD. Furthermore, the interplay of an epigenetic hallmark, such as DNA methylation, as negatively correlated with the H3K4me3 mark may explain the hypermethylation in the hypermutated samples.^32^ Meanwhile, frequent depletion of the H3K36 methyltransferase SETD2 (21%) and H3K4 methyltransferase SETD1B (34%) in CRC and H3K4 methyltransferase SETD1A (21%) in STAD, which are all associated with gene transcription, elongation, or alternative splicing,^33^, ^34^, ^35^, ^36^ may also play a role in remodeling consistent hypermutated expression and DNA methylation patterns.

A remarkably favorable prognosis has been proposed in MMR‐deficient colorectal tumors.^37^ Multiple mechanisms may be underlying this proposal. Hypermutated CRC patients are more prevalent in stage I/II but are rare in advanced stages (*P* < 0.001, *χ* ^2^ test). This result also holds true in CRC samples collected at Zhejiang University Cancer Institute. Sequencing (71 whole exome sequencing, 10 whole genome sequencing and 257 target sequencing) of 338 pairs of matched fresh frozen colorectal cancer tissue and adjacent normal tissue samples, 45 were hypermutated (stage I: 4, II: 27, III: 13, IV: 1, unpublished data). These results suggest that hypermutated CRCs are resistant to tumor metastasis and may partially account for the better survival in hypermutated patients. In addition, better survival may also be linked to neoantigen enrichment. Recently, Germano et al^13^, found that inactivation of MMR triggers neoantigen generation and impairs tumor growth. As mentioned earlier, frequent mutation of chromatin remodeling genes by MSI‐H in hypermutated subgroups play a vital role in determining consistent expression and methylation patterns. Thus, we also want to know if the mutation of chromatin remodeling genes will be accompanied by a better prognosis. As direct mutation evidence suggests, somatic mutations in chromatin‐regulating genes *MLL1˜3* and *ARID1A* in pancreatic cancer patients are associated with improved survival.^38^ In addition, this association may also hold true in CRC and STAD.

Taking advantage of the consistent expression and DNA methylation patterns between CRC and STAD observed in this study could lead to the use of widely applicable targeted therapies. For the first time, in 2017, the U.S. Food and Drug Administration (FDA) approved a cancer drug, *Keytruda*, for the treatment of any solid tumor that displays a high MSI, regardless of the organ site. The up‐regulated genes identified in this study may also be potential drug targets that are not specific to the organ site of the tumor and are not restricted to only neoantigens accompanied by mutation. Recently, Lin et al^39^ found that adenocarcinomas and squamous cell carcinomas reveal molecular similarities that span classic anatomic boundaries via the comparison of their transcriptomes. This finding further weakens the impact of organ‐driven medical decision making in future cancer therapy.

*BRAF* and *KRAS* mutations were significantly associated with shorter disease‐free survival (DFS) and overall survival (OS) in patients with microsatellite‐stable tumors but not in patients with MSI tumors.^40^ This finding suggests that the mutation of many other genes counteract the negative effect offered by *BRAF* and *KRAS* mutations. An MSI test may be more important than single or dual gene *BRAF* and *KRAS* detection. It is thus reasonable and a priority to stratify patients into hypermutated and non‐hypermutated subgroups but not based on organ site for better treatment in future cancer therapy. Identification of the expression or methylation markers that can predict clinical outcomes of hypermutated patients are beneficial for adopting reasonable treatment strategy. In addition, markers identified in this study are probably overlooked by previous hyper/non‐hyper pool analysis. It is thus necessary and important to identify biomarkers independently and according to mutational subtypes such as hypermutated and non‐hypermutated states.

## CONFLICT OF INTEREST

The authors confirm that there are no conflicts of interest.
