# Supplementary material for: Deciphering molecular properties of hypermutated gastrointestinal cancer
Source: J Cell Mol Med. 2018 Oct 31;23(1):370–9. doi: 10.1111/jcmm.13941 (PMC6307802; doi:10.1111/jcmm.13941)
Supplement: Supplementary file 1 [file JCMM-23-370-s001.pptx]

## Slide 1
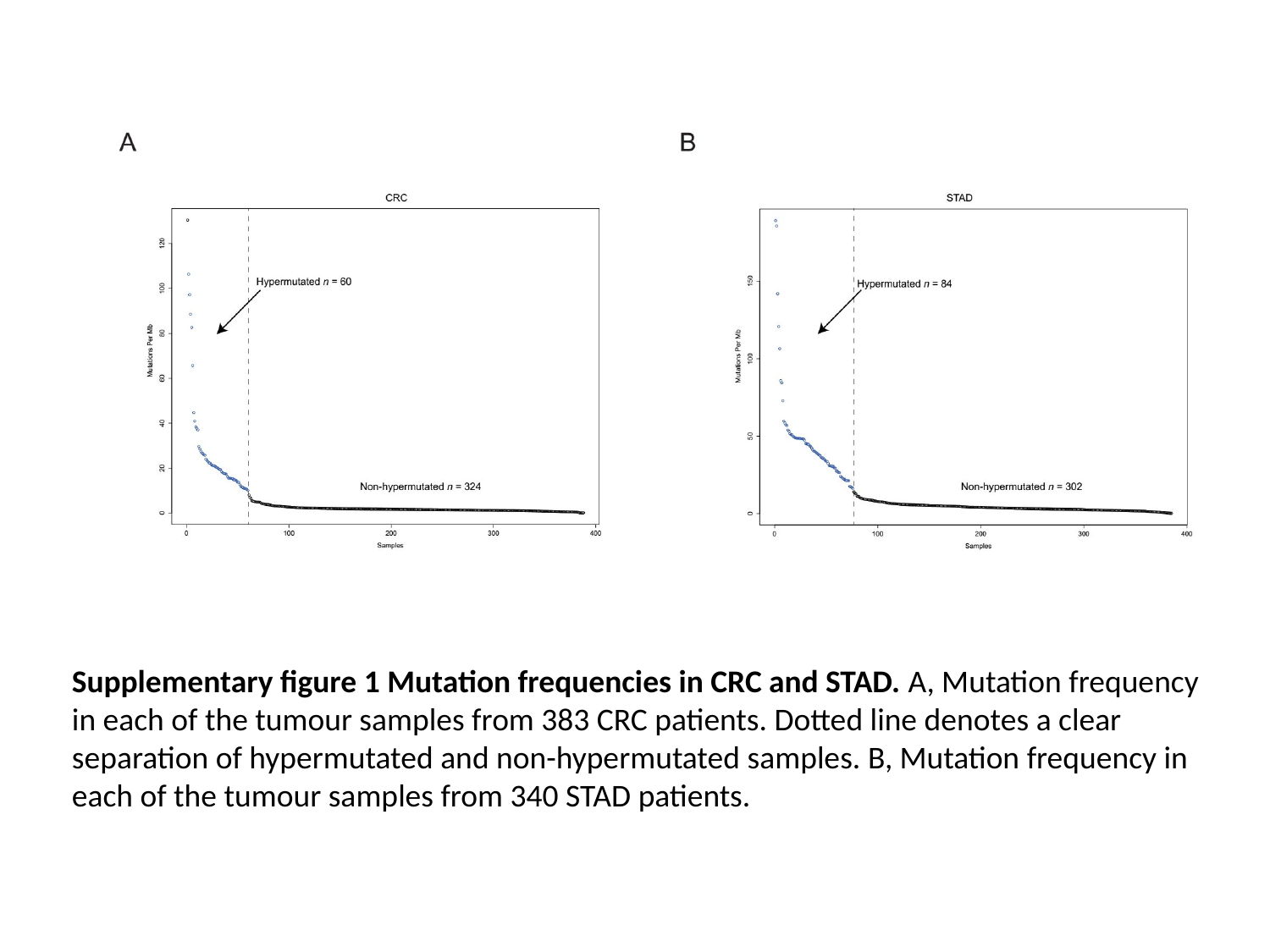

Supplementary figure 1 Mutation frequencies in CRC and STAD. A, Mutation frequency in each of the tumour samples from 383 CRC patients. Dotted line denotes a clear separation of hypermutated and non-hypermutated samples. B, Mutation frequency in each of the tumour samples from 340 STAD patients.

## Slide 2
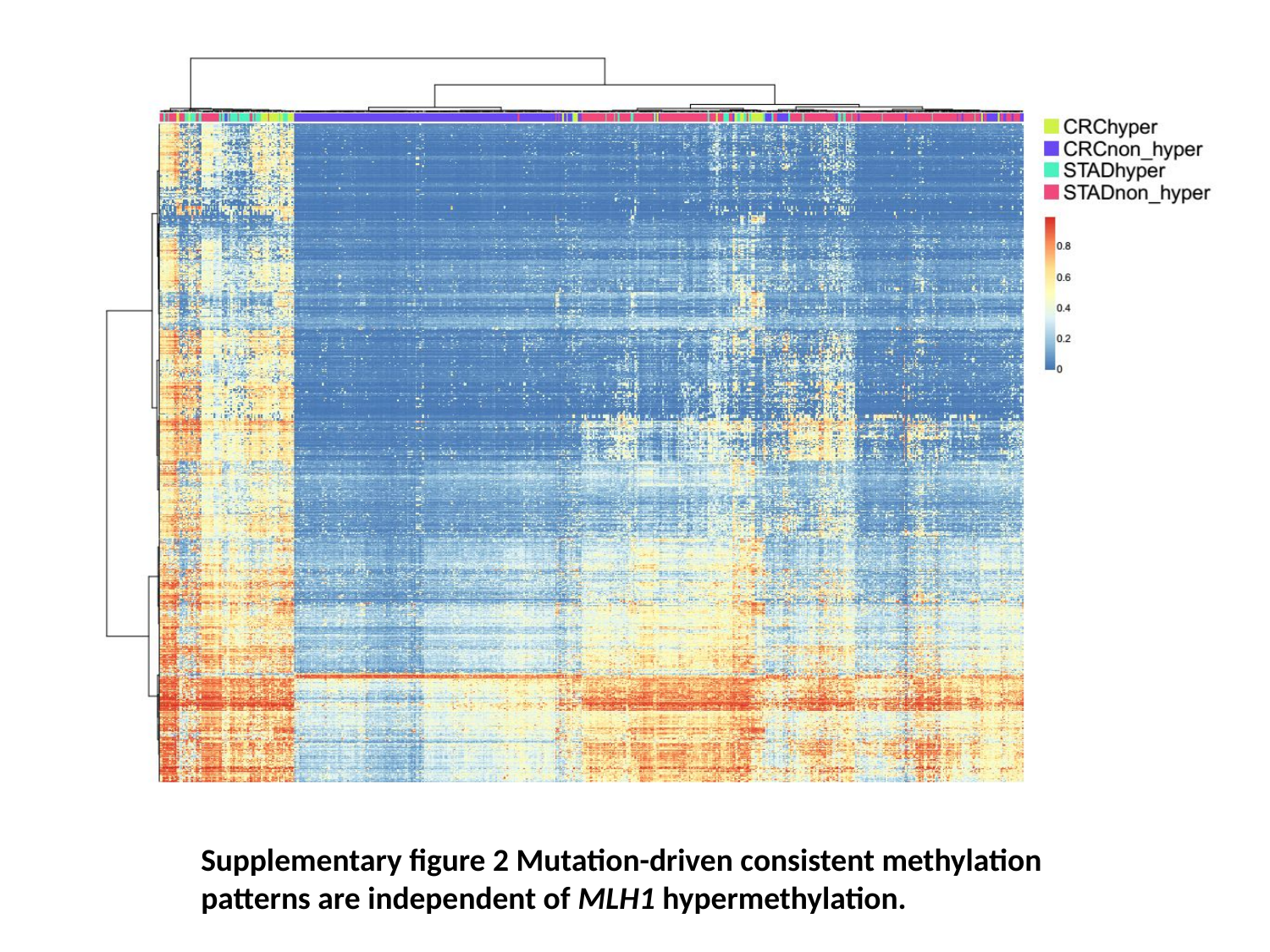

Supplementary figure 2 Mutation-driven consistent methylation patterns are independent of MLH1 hypermethylation.

## Slide 3
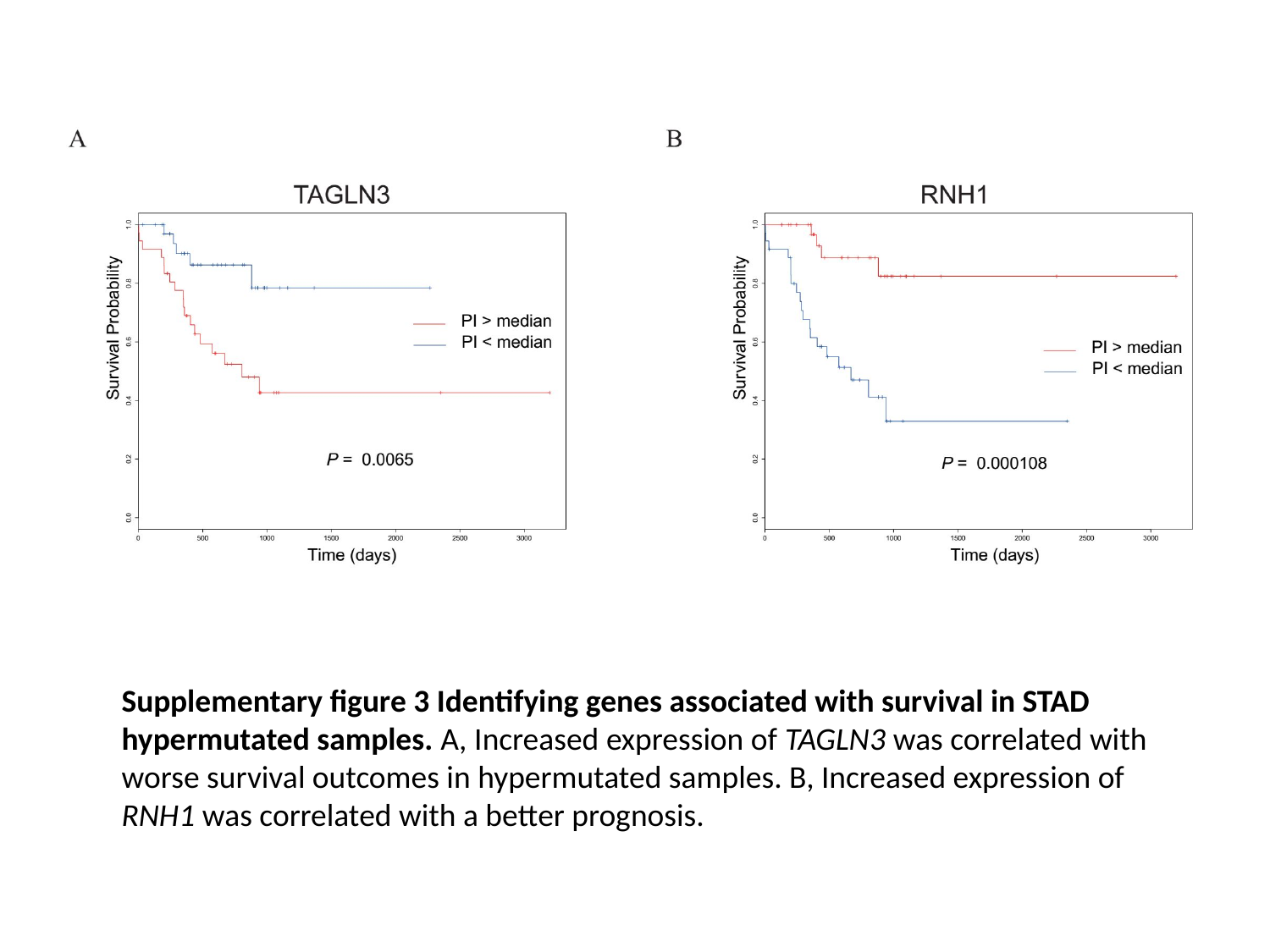

Supplementary figure 3 Identifying genes associated with survival in STAD hypermutated samples. A, Increased expression of TAGLN3 was correlated with worse survival outcomes in hypermutated samples. B, Increased expression of RNH1 was correlated with a better prognosis.
